# CVm6A: A Visualization and Exploration Database for m^6^As in Cell Lines

**DOI:** 10.3390/cells8020168

**Published:** 2019-02-17

**Authors:** Yujing Han, Jing Feng, Linjian Xia, Xin Dong, Xinyang Zhang, Shihan Zhang, Yuqi Miao, Qidi Xu, Shan Xiao, Zhixiang Zuo, Laixin Xia, Chunjiang He

**Affiliations:** 1 School of Basic Medical Sciences, Wuhan University, Wuhan 430071, Hubei, China; hanyujing@whu.edu.cn (Y.H.); linjianxia@whu.edu.cn (L.X.); dongxin@whu.edu.cn (X.D.); zhangxinyang@whu.edu.cn (X.Z.); 13699447096@163.com (S.Z.); myq_victoria@163.com (Y.M.); xuqidi01@outlook.com (Q.X.); 2 Hubei Province Key Laboratory of Allergy and Immunology, Wuhan 430071, Hubei, China; 3 Hubei Provincial Key Laboratory of Developmentally Originated Disease, Wuhan 430071, Hubei, China; 4 School of Computer Science, Wuhan University, Wuhan 430072, Hubei, China; gfeng@whu.edu.cn; 5 Department of Developmental Biology, School of Basic Medical Sciences, Southern Medical University, Guangzhou 510515, China; asdfg@smu.edu.cn; 6 Sun Yat-sen University Cancer Center, State Key Laboratory of Oncology in South China, Collaborative Innovation Center for Cancer Medicine, Sun Yat-sen University, Guangzhou 510060, China; zuozhx@sysucc.org.cn

**Keywords:** N6-methyladenosine, cell line, m^6^A, visualization

## Abstract

N6-methyladenosine (m^6^A) has been identified in various biological processes and plays important regulatory functions in diverse cells. However, there is still no visualization database for exploring global m^6^A patterns across cell lines. Here we collected all available MeRIP-Seq and m^6^A-CLIP-Seq datasets from public databases and identified 340,950 and 179,201 m^6^A peaks dependent on 23 human and eight mouse cell lines respectively. Those m^6^A peaks were further classified into mRNA and lncRNA groups. To better understand the potential function of m^6^A, we then mapped m^6^A peaks in different subcellular components and gene regions. Among those human m^6^A modification, 190,050 and 150,900 peaks were identified in cancer and non-cancer cells, respectively. Finally, all results were integrated and imported into a visualized cell-dependent m^6^A database CVm6A. We believe the specificity of CVm6A could significantly contribute to the research for the function and regulation of cell-dependent m^6^A modification in disease and development.

## 1. Introduction

As one important post-transcriptional modification, N6-methyladenosine (m^6^A) was largely discovered by high throughput sequencing in recent years [1,2,3]. m^6^A was identified with consensus sequence surrounding m^6^A site RRACH (R=G or A, H=A, C or U) and conserved in human, mouse, chimpanzee and even in plants [1,4,5]. m^6^A was also found to exist in bacterial and archaeal species [6]. The abundance of m^6^A is reported as being correlated with evolutionarily conserved region of genome [2]. m^6^A modification was a reversible status mediated by methyltransferases METTL3/ METTL14/ WTAP complex [7], demethylases FTO/ALKBH5 [2,8] and recognized by m^6^A binding proteins YTH (YT521-B homology) domain family/HNRNPA2B1 [9,10], which were called writer, eraser and reader, respectively.

m^6^A can regulate the multiple biological functions in spatial and temporal [11]. m^6^A methyltransferase complex controls the neuronal functions and fine-tuning sex determination in Drosophila [12]. m^6^A also acts as a regulator at molecular switches in murine naive pluripotency for proper lineage priming and differentiation [13]. The existence of m^6^A in lncRNA XIST mediated the gene silencing on X chromosome. Knockdown of m^6^A methytransferase METTL3 can impair XIST-mediated gene silencing [14]. m^6^A RNA can recruit DNA polymerase k (Pol k) to facilitate repairing of ultraviolet-induced DNA damage [15]. Furthermore, m^6^A could alter RNA structure to affect RNA-protein interactions in cells [16]. The m^6^A-driven gene network was already constructed and the dynamic interactions between m^6^A related methyltransferases and demthylases were established [17]. The deficiency of m^6^A modification led to various diseases, such as obesity, cancer, type 2 diabetes mellitus, infertility and developmental arrest, etc. [18].

In previous researches, m^6^A was discovered mainly located near stop codons, large internal exons and 3’UTR (3’-Untranslated region), as well as in CDS (Coding sequence), transcriptional start sites and intron [1,2,19]. Dynamic m^6^A modification could affect translation status and lifetime of mRNA in Hela [20]. Several lncRNAs also hosted m^6^A modification [1,2] and long intergenic noncoding RNAs (lincRNAs) established significantly higher level than mRNAs in B-cell lymphoblastoid cell line GM12878 [21]. In CD4T, m^6^A modification happened on HIV-1 RNA could regulate viral infection [22].

Though m^6^A patterns were analyzed in different cells independently, the global patterns across those cells were not well summarized. Several databases collected and detected m^6^A from public datasets, such as RMBase [23] and MeT-DB [24]. However, RMBase and MeT-DB were not focused on cell-dependent m^6^A. For examples, MeT-DB only included m^6^A datasets from a portion of wild type cell lines, and RMBase included m^6^A sites from various samples without indicating the cell sources. To better understand the function of m^6^A in cellular biological processes, a more specific database is needed for exploring and comparing the distribution and patterns of m^6^A in different cell lines. Here, using latest public datasets, we collected MeRIP-Seq and m^6^A-CLIP-Seq datasets from 23 human cell lines and eight mouse cell lines from previous work, and inspected the global patterns of m6As across those cell lines, including the distribution and abundance of m^6^A modification in lncRNA or mRNA, different subcellular location and gene regions. The m^6^A patterns from cancer or non-cancer cell lines were also classified. Moreover, validated m^6^A sites from previous experiments were also collected and summarized. All results were imported into a cell-dependent m^6^A database CVm6A (http://gb.whu.edu.cn:8080/CVm6A) providing a visualization interface for searching and comparing the m^6^A patterns in different cell lines, which could contribute to the function and regulation research of m^6^A in disease and development.

## 2. Data Collection and Database Content

### 2.1. Cell Line Samples in CVm6A

Previous studies showed that MeRIP-Seq (Methylated RNA Immunoprecipitation sequencing) [20], miCLIP-Seq (m^6^A individual-nucleotide-resolution cross-linking and immunoprecipitation sequencing) [25] and PA-m^6^A-Seq (Photo-crosslinking-assisted m^6^A-seq) [26] could be used for detecting m^6^A modification in transcriptomic level. Therefore, we collected all available MeRIP-Seq, miCLIP-Seq and PA-m^6^A-Seq datasets with total RNA or PolyA enriched library construction from NCBI GEO database (http://www.ncbi.nlm.nih.gov/GEO). In total, 47 samples from 23 human cell lines and 22 samples from 8 mouse cell lines were collected (Table S1).

### 2.2. Identification of Cell m^6^A Peaks

**Figure 1:**
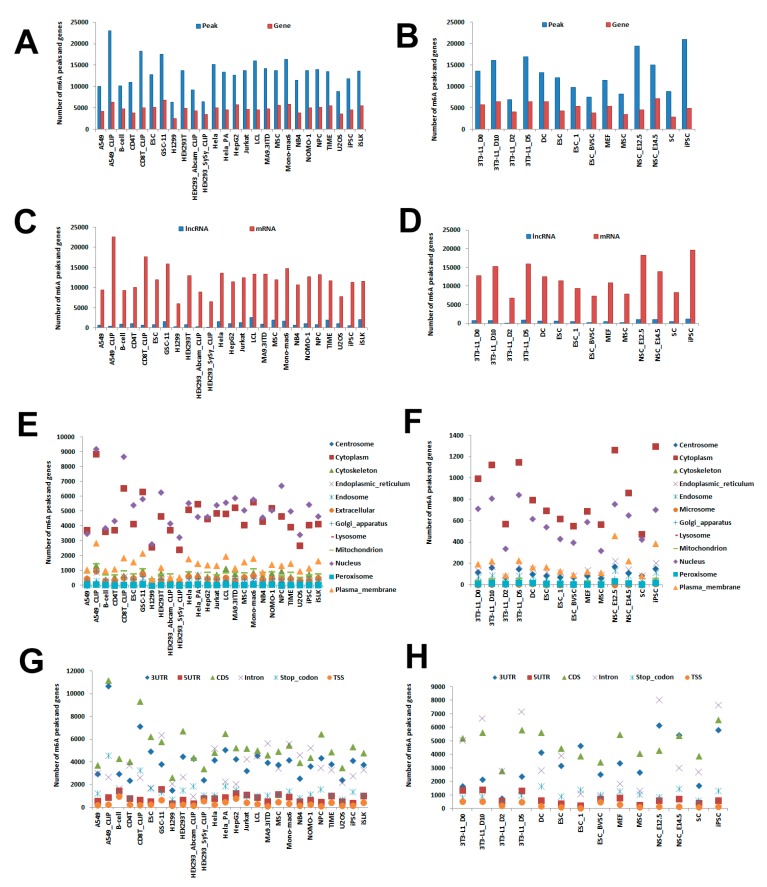
Statistics of m^6^A patterns in CVm6A. (**A**) Number of m^6^A peaks and genes in human cell lines. (**B**) Number of m^6^A peaks and genes in mouse cell lines. (**C**) Number of m^6^A peaks distributed in lncRNA or mRNA in human cell lines. (**D**) Number of m^6^A peaks distributed in lncRNA or mRNA in mouse cell lines. (**E**) Number of m^6^A peaks distributed in 12 subcellular components in human cell lines. (**F**) Number of m^6^A peaks distributed in 12 subcellular components in mouse cell lines. (**G**) Number of m^6^A peaks distributed in 6 gene regions: TSS (Transcription start site), 5’UTR, CDS, Stop codon, 3’UTR and intron in human cell lines. (**H**) Number of m^6^A peaks distributed in 6 gene regions: TSS, 5’UTR, CDS, Stop codon, 3’UTR and intron in mouse cell lines.

For MeRIP-Seq datasets, both reads from IP (Immunoprecipitation) and Input samples were mapped to human (hg38 version) and mouse (mm10 version) genome separately via Hisat2 [27]. Mapped reads with MAPQ <30 were filtered by samtools [28], and removed PCR duplicates using Picard (http://broadinstitute.github.io/picard, v2.16.0). Then m^6^A peaks were called and enrichment score of each peak was calculated by MeTPeak [29]. m^6^A sites from miCLIP-Seq and PA-m^6^A-Seq were collected from previous works [19,25,26]. Gene annotation of GENCODE (GRCh38 release 28 and GRCm38 release M20) including 35,048 human genes and 31,237 mouse genes were used to annotate m^6^A sites or peaks. Detailed pipeline was included in Supplemental Method. In all cell lines, total 340,950 m^6^A peaks from 16,950 human genes and 179,201 m^6^A peaks from 14,360 mouse genes were identified. In human cell lines, we retrieved 6345 (H1299) ~ 23,052 (A549) m^6^A peaks, and 2562 (H1299) ~ 6838 (GSC-11) genes with m^6^A modification (Figure 1A). In mouse cell lines, 6833 (3T3-L1) ~ 20,892 (iPSC) m^6^A peaks and 2882 (SC) ~ 7125 (NSC) genes with m^6^A modification were identified (Figure 1B).

### 2.3. Prediction of m^6^A lncRNA and mRNA

All m^6^A peaks were mapped to mRNA and lncRNA using GENCODE gene annotation (GRCh38 release 28 and GRCm38 release M20) [30] via Bedtools [31]. To view the similarities and differences of m^6^A modification in lncRNA and mRNA, those all m^6^A genes were separated into lncRNA or mRNA groups. In human cell lines, there were 225 (HEK293) ~ 2627 (LCL) peaks from lncRNA, while 6044 (H1299) ~ 22,630 (A549) peaks were from mRNA (Figure 1C). We also checked the enrichment score of those m^6^A peaks from lncRNA and mRNA. The average enrichment scores of lncRNA were from 2.37 (U2OS) ~ 9.55 (ESC), while in mRNAs, the scores were from 2.36 (U2OS) ~ 11.15 (iPSC) (Figure S1A). In mouse cell lines, there were 101 (3T3-L1) ~ 1,243 (iPSC) peaks from lncRNA, while 6732 (3T3-L1) ~ 19,649 (iPSC) peaks were from mRNA (Figure 1D). The average enrichment scores of lncRNA and mRNA from mouse cell lines were also established (Figure S1B).

### 2.4. Prediction of Subcellular Location

To view the location of m^6^A in subcellular component, we acquired the subcellular location information of genes from public database Hum-mPloc 3.0 [32] and Euk-mPLoc [33] and classified m^6^A peaks into subcellular components according to their annotated genes. Total 309,137 peaks were located into different components in all cell lines. Across human cell lines, 2439 (HEK293) ~ 8866 (A549) of peaks were located in Cytoplasm, 2772 (H1299) ~ 9195 (A549) of peaks located in Nucleus and 447 (H1299) ~ 2878 (A549) of m^6^A peaks located in Plasma membrane. Other peaks were distributed in other subcellular location: Centrosome, Cytoskeleton, Endoplasmic reticulum, Endosome, Golgi apparatus, Lysosome, Mitochondrion, Peroxisome, and Extracellular (Figure 1E). The average enrichment scores of those peaks from different components were also calculated (Figure S1C). Similar distributions were observed in mouse cell lines (Figure 1F and Figure S1D).

### 2.5. Prediction of Gene Regions

Previous works revealed m^6^A modification were not uniform on different gene regions [1,2,20]. In CVm6A, we separated all annotated genes into 6 regions: TSS, 5′UTR, CDS, Stop codon, 3′UTR and Intron and located m^6^A peaks in these regions. The middle site of each peak from MeRIP-Seq or the precise m^6^A site from m^6^A-CLIP-Seq was used for the localization. For TSS and stop codon, a 200 bp window (±100 bp surrounding the coordinates) were allowed to locate the m^6^A site according to previous work [1,2]. Across human cell lines, 2668 (H1299) ~ 11,209 (A549) of m^6^A peaks were distributed in CDS, while 1517 (H1299) ~ 10,706 (A549) were located in 3′UTR and 710 (CD8T) ~ 4608 (A549) were distributed in Stop codon (Figure 1G). We also checked the average enrichment scores distributed in those regions. In all cell lines, stop codon had average scores from 2.34 (U2OS) to 12.5 (iPSC), while the average scores in CDS were 2.41 (U2OS) ~ 11.7 (iPSC) and the average scores in 3′UTR were 2.32 (U2OS) ~ 11.6 (iPSC) (Figure S1E). The m^6^A distribution of gene regions in mouse cell lines was also established (Figure 1H and Figure S1F).

### 2.6. Classification of Cancer and Non-Cancer m^6^A

To view the association of m^6^A and diseases, all human m^6^A peaks were classified into cancer and non-cancer groups. Overall, 190,050 m^6^A peaks from 14,628 genes were identified in 12 cancer cell lines and 150,900 peaks from 14,346 genes were identified in 11 non-cancer cell lines (Figure S1G).

### 2.7. Validated m^6^A Sites

Previous research had validated several m^6^A modification in cell lines. To enhance the usability of CVm6A, we collected those m^6^A sites from m^6^A-RIP or m^6^A-CLIP experiment from previous literatures. Totally, CVm6A contains validated m^6^A modification in 96 genes, which were identified in 25 cell lines from human, mouse, zebrafish and Drosophila (Table S2).

## 3. Database Organization and Web Interface

**Figure 2:**
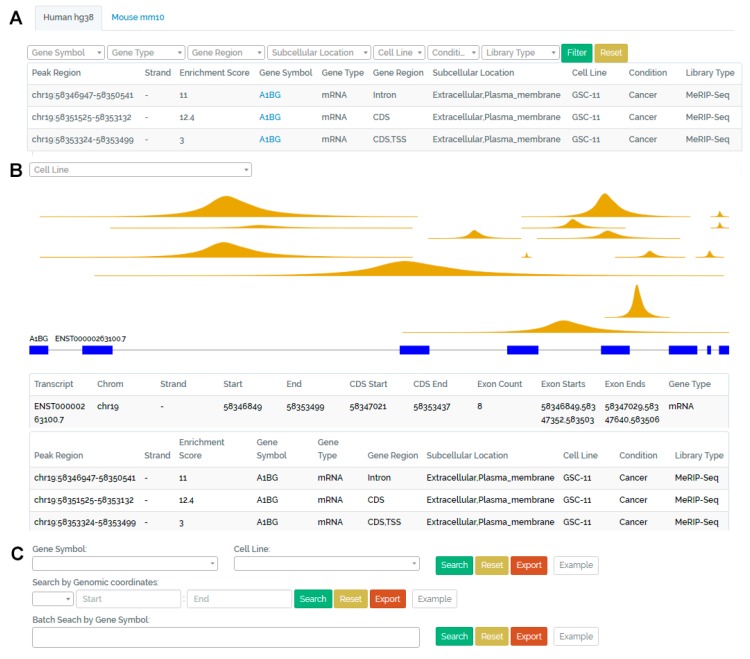
Overview of CVm6A. (**A**) Browse page. All m^6^A peaks in this page can be filtered by gene symbol, gene type, gene region, subcellular location, cell line, condition and library type. The peak region and gene symbol are linked to the visualization page. (**B**) Visualization page. All m^6^A peaks in a selected gene are displayed with dark yellow color. Peaks in selected cell lines are displayed with brown color. Annotated gene and transcripts structure are displayed in blue box (exon) and gray line (intron). Coordinates of annotated transcripts and table with detailed information corresponding to these peaks are displayed below. (**C**) Search page. Users can search all m^6^A peaks in a special gene or cell line. The Search function by genomic coordinates supports fuzzy search of all peaks surrounding the input genomic region. Batch search by gene symbol is also provided.

All the analyzed results, including peak region, gene type, gene region, subcellular location, conditions and library types associated with m^6^A peaks were integrated into a set of interactive MySQL tables. Laravel–an open-source web framework based in PHP (https://laravel.com) and JavaScript library were used to construct the CVm6A database. The web interface of CVm6A is summarized in Figure 2.

### 3.1. Browse Page

On this page, users can browse all m^6^A peaks from 23 human and eight mouse cell lines. All information, including the peak region, strand, enrichment score, gene symbol, gene type (mRNA/lncRNA), gene region (CDS, 3’UTR, et al.), subcellular location (plasma_membrane, nucleus, et al.), cell line, condition (cancer/non-cancer) and library type for each peak are displayed in the table (Figure 2A). Peak region and gene symbol are linked to the visualization page of m^6^A peaks located in this gene (Figure 2B).

### 3.2. Visualization Page

On this page, users can view all m^6^A peaks distributed in selected annotated gene. All peaks in current gene are displayed with dark yellow color. In the top left corner, user can also select special cell line in the search box. All peaks in the selected cell line are displayed with brown color both in the figure and table. The summit of each peak is determined according to the enrichment score. The gene structure with exon (blue box) and intron (gray line) is displayed below peak figures. If the selected gene has more than one transcript, all transcripts are displayed. The relative location of each peak and the annotated gene are placed according to genomic coordinates. Mouse hover over on or click each peak can display the peak region, enrichment score, gene region, subcellular location, cell line, condition and library type of this peak. The table below the figures includes the coordinates of annotated transcripts and detailed information of all peaks in the selected gene (Figure 2B).

### 3.3. Search Page

On this page, users can search m^6^A peaks by gene symbol, cell line or genomic coordinates. While users select gene symbol or cell line, all peaks in this gene or cell line will be displayed in tables below and can be exported into files. Batch search by gene symbol is also provided. The search function by genomic coordinates supports fuzzy search. All peaks surrounding the input coordinates will be displayed (Figure 2C).

## 4. Summary and Future Directions

CVm6A collects available MeRIP-Seq and m^6^A-CLIP-Seq datasets in human and mouse cell lines, and provides a visualized m^6^A database to benefit functional studies of m^6^A in cell lines. Those samples include the most frequently used cell lines in previous researches. For example, users working on stem cells could explore the m^6^A modification in ESC, MSC, NPC and iPSC and can compare the distribution of m^6^A peaks in nucleus and other subcellular components, as well as 3′UTR and other gene regions. Users working on the immune cell lines could inspect the distribution in CD4T, CD8T and LCL. Moreover, more than ten cancer cell lines are included in CVm6A, which allow researchers to study the potential function of m^6^A in cancers. CVm6A also predicts the enrichment score of each peak, which allow users to check the abundance of m^6^A. Due to the limited m^6^A-Seq datasets for total RNA, only two total RNA datasets are included in the current version, which cannot thoroughly establish the distribution of m^6^A on lncRNA and other non-polyA RNAs. We will update CVm6A when more sequencing data from other library types becomes available.

## Supplementary Materials

The supplementary materials are available online http://www.mdpi.com/2073-4409/8/2/168/s1.
