# Supplementary material for: CVm6A: A Visualization and Exploration Database for m6As in Cell Lines
Source: Cells. 2019 Feb 17;8(2):168. doi: 10.3390/cells8020168 (PMC6406471; doi:10.3390/cells8020168)
Supplement: Supplementary file 1 [file cells-08-00168-s001.pdf]

SUPPLEMENTAL MATERIALS

CVm6A: A visualization and exploration database for m⁶As in cell lines

Yujing Han[#1,2,3], Jing Feng[#4], Linjian Xia[#1,2,3], Xin Dong[1], Xinyang Zhang[1], Shihan Zhang[1], Yuqi Miao[1], Qidi Xu[1], Shan Xiao[5], Zhixiang Zuo[6], Laixin Xia[*5], Chunjiang He[*1,2,3]

# These authors contributed equally

Supplemental Figure Legends

Figure S1. Statistics of m^{6}A patterns in CVm6A. A. Average m^{6}A enrichment score in lncRNA and mRNA in human cell lines. **B.** Average m^{6}A enrichment score in lncRNA and mRNA in mouse cell lines. **C.** Average m^{6}A enrichment score in 12 subcellular components in human cell lines. **D.** Average m^{6}A enrichment score in 12 subcellular components in mouse cell lines. **E.** Average m^{6}A enrichment score in 6 gene regions in human cell lines. **F.** Average m^{6}A enrichment score in 6 gene regions in mouse cell lines. **G**. Number of all m^{6}A peaks and genes with m^{6}A modification in human cancer and non-cancer cell lines.

Supplemental Method

Pipeline for running meRIP-Seq datasets

1. Quality Control

Quality control for the sequencing reads is suggested before mapping to genome.

1.1 Run FastQC (http://www.bioinformatics.babraham.ac.uk/projects/fastqc/)

```
>>>fastqc Sample.R1.fq.gz Sample.R2.fq.gz
```

1.2 Remove adapter and low quality bases

```
>>>trim_galore -q 20 --phred33 --stringency 3 --length 20 -e 0.1 -paired Sample.R1.fq
Sample.R2.fq --gzip -o Sample
```

1.3 Check the quality again

```
>>>fastqc Sample.R1_trimmed.fq.gz Sample.R2_trimmed.fq.gz
```

2. Map to genome by Hisat2 [1]

2.1 Generate genome indices

```
>>>hisat2-build -p NumberOfThreads  /path/to/genome/fasta /path/to/your/index/hisat2-
index
```

2.2 Mapping

```
>>>hisat2 -x /path/to/your/index/hisat2-index
 -p NumberOfThreads -1 Sample.R1.fq -2 Sample.R2.fq  -S Sample.sam
```

3. Get bam file by samtools [2]

```
>>>samtools view -bhS -q 30 -@ NumberOfThreads  Sample.sam >Sample.bam
>>>samtools sort -o Sample_sorted.bam -T sorted -@ NumberOfThreads  Sample.bam
```

4. Running Picard (http://broadinstitute.github.io/picard**)**

```
>>>picard.jar MarkDuplicates I=Sample_sorted.bam O=Sample_last.bam
REMOVE_DUPLICATES=true ASSUME_SORT_ORDER=coordinate
CREATE_INDEX=true VALIDATION_STRINGENCY=SILENT
M=Sample_marked_dup_metrics.txt
```

5. Peak calling using MeTPeak [3]

```
library(MeTPeak)
gtf <- system.file('extdata','example.gtf',package='MeTPeak')
ip1 <- system.file('extdata','IP1.bam',package='MeTPeak')
ip2 <- system.file('extdata','IP2.bam',package='MeTPeak')
ip3 <- system.file('extdata','IP3.bam',package='MeTPeak')
input1 <- system.file('extdata','Input1.bam',package='MeTPeak')
input2 <- system.file('extdata','Input2.bam',package='MeTPeak')
input3 <- system.file('extdata','Input3.bam',package='MeTPeak')
IP_BAM <- c(ip1,ip2,ip3)
INPUT_BAM <- c(input1,input2,input3)
metpeak(GENE_ANNO_GTF=gtf,IP_BAM = IP_BAM,INPUT_BAM = INPUT_BAM,
    EXPERIMENT_NAME="example")
```

REFERENCE

1. Pertea, M.; Kim, D.; Pertea, G.M.; Leek, J.T.; Salzberg, S.L. Transcript-level expression analysis of rna-seq experiments with hisat, stringtie and ballgown. *Nat Protoc* **2016**, *11*, 1650-1667.
2. Li, H.; Handsaker, B.; Wysoker, A.; Fennell, T.; Ruan, J.; Homer, N.; Marth, G.; Abecasis, G.; Durbin, R.; Genome Project Data Processing, S. The sequence alignment/map format and samtools. *Bioinformatics* **2009**, *25*, 2078-2079.
3. Cui, X.; Meng, J.; Zhang, S.; Chen, Y.; Huang, Y. A novel algorithm for calling mrna m6a peaks by modeling biological variances in merip-seq data. *Bioinformatics* **2016**, *32*, i378-i385.

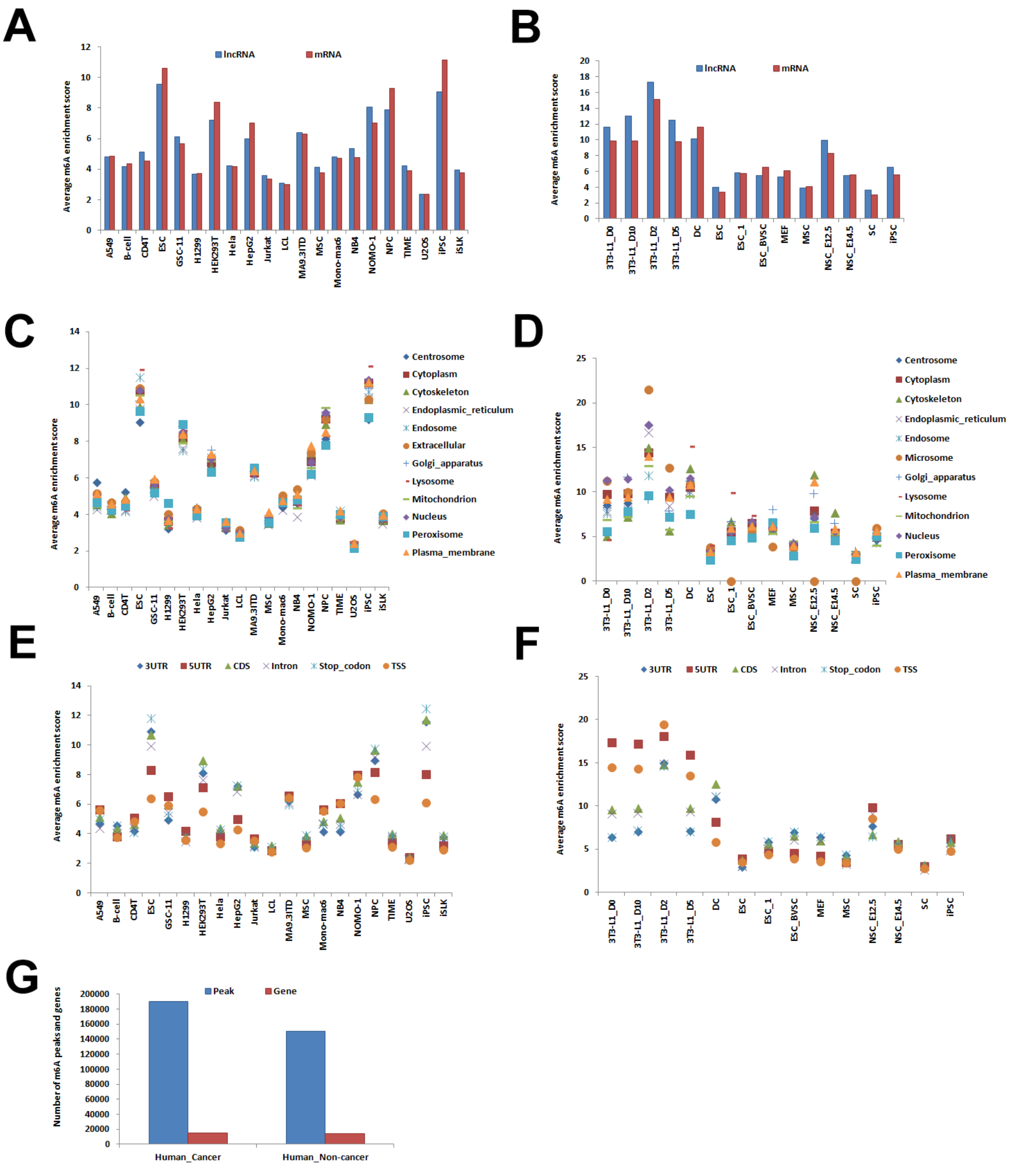

Figure S1. Statistics of m6A patterns in CVm6A. **A.** Average m6A enrichment score in. lncRNA and mRNA in human cell lines. **B.** Average m6A enrichment score in lncRNA and mRNA in mouse cell lines. **C.** Average m6A enrichment score in 12 subcellular components in human cell lines. **D.** Average m6A enrichment score in 12 subcellular components in mouse cell lines. **E.** Average m6A enrichment score in 6 gene regions in human cell lines. **F.** Average m6A enrichment score in 6 gene regions in mouse cell lines. **G**. Number of all m6A peaks and genes with m6A modification in human cancer and noncancer cell lines.

Table S1. Cell line samples with MeRIP-Seq and miCLIP-Seq in CVm6A

line and treat	Technology	PubmedID	Study	IP Sample	nput sampl	Information	Library type	Condition	Species
A549	MeRIP-Seq	Citation missing	GSE76367	GSM198225	GSM198225	Lung cancer	PolyA+	Cancer	Human
A549_CLIP	miCLIP-Seq	26404942	GSE71154	GSM1828596		Lung cancer	PolyA+	Cancer	Human
B-cell	MeRIP-Seq	Citation missing	GSE113798	GSM311958	GSM311957	human B-lyn	PolyA+	Non-cancer	Human
CD4T	MeRIP-Seq	27371828	GSE85724	GSM228321	GSM228320	Primary CD4	PolyA+	Non-cancer	Human
CD8T_CLIP	miCLIP-Seq	26404942	GSE71154	GSM1828594		CD8+ T cell	PolyA+	Non-cancer	Human
ESC	MeRIP-Seq	24981863	GSE55572	GSM133939	GSM133939	human embr	PolyA+	Non-cancer	Human
GSC-11	MeRIP-Seq	28344040	GSE87515	GSM233297	GSM233297	glioblastoma	PolyA+	Cancer	Human
H1299	MeRIP-Seq	Citation missing	GSE76367	GSM198226	GSM198226	Lung cancer	PolyA+	Cancer	Human
HEK293T	MeRIP-Seq	24981863	GSE55572	GSM133940	GSM133940	Human Emb	PolyA+	Non-cancer	Human
HEK293_SyS	miCLIP-Seq	26121403	GSE63753	GSM1556678		embryonic ki	total RNA	Non-cancer	Human
HEK293_Abc	miCLIP-Seq	26121403	GSE63753	GSM1556679		embryonic ki	total RNA	Non-cancer	Human
Hela	MeRIP-Seq	24284625	GSE46705	GSM113502	GSM113501	cervical canc	PolyA+	Cancer	Human
Hela_PA	PA-m^{6}A-seq	25491922	GSE54921	GSM1326564,GSM13265		cervical canc	PolyA+	Cancer	Human
HepG2	MeRIP-Seq	22575960	GSE37005	GSM908337	GSM908341	human liver	PolyA+	Cancer	Human
iPSC	MeRIP-Seq	24981863	GSE54365	GSM133940	GSM133940	OKMS induc	PolyA+	Non-cancer	Human
iSLK	MeRIP-Seq	29109479	GSE93676	GSM246034	GSM246034	doxycycline-	PolyA+	Non-cancer	Human
Jurkat	MeRIP-Seq	27371828	GSE85724	GSM228321	GSM228320	immortalized	PolyA+	Cancer	Human
LCL	MeRIP-Seq	28052920	GSE70299	GSM172334	GSM172334	lymphoblast	PolyA+	Non-cancer	Human
MA9.3ITD	MeRIP-Seq	29249359	GSE87190	GSM232429	GSM232429	acute monoc	PolyA+	Cancer	Human
Mono-mac6	MeRIP-Seq	28017614	GSE76414	GSM201045	GSM201045	acute monoc	PolyA+	Cancer	Human
MSC	MeRIP-Seq	29109479	GSE93676	GSM246035	GSM246035	Mesenchym	PolyA+	Non-cancer	Human
NB4	MeRIP-Seq	29290617	GSE97408	GSM256401	GSM256401	acute myeloi	PolyA+	Cancer	Human
NOMO-1	MeRIP-Seq	29249359	GSE87190	GSM232429	GSM232429	acute monoc	PolyA+	Cancer	Human
NPC	MeRIP-Seq	24981863	GSE55572	GSM133939	GSM133939	human neur	PolyA+	Non-cancer	Human
TIME	MeRIP-Seq	29109479	GSE93676	GSM246035	GSM246035	immortalized	PolyA+	Cancer	Human
U2OS	MeRIP-Seq	24209618	GSE48037	GSM116613	GSM116613	human osteo	PolyA+	Cancer	Human
3T3-L1_D0	MeRIP-Seq	25412662	GSE53244	GSM128848	GSM128847	D0 day Mous	PolyA+	Non-cancer	Mouse
3T3-L1_D10	MeRIP-Seq	25412662	GSE53244	GSM128848	GSM128848	D10 day Mou	PolyA+	Non-cancer	Mouse
3T3-L1_D2	MeRIP-Seq	25412662	GSE53244	GSM128847	GSM128847	D2 day Mous	PolyA+	Non-cancer	Mouse
3T3-L1_D5	MeRIP-Seq	25412662	GSE53244	GSM128848	GSM128848	D5 day Mous	PolyA+	Non-cancer	Mouse
DC	MeRIP-Seq	24981863	GSE54365	GSM133947	GSM133947	Dendritic cel	PolyA+	Non-cancer	Mouse
ESC	MeRIP-Seq	25683224	GSE52125	GSM126005	GSM126005	mouse embr	PolyA+	Non-cancer	Mouse
ESC_1	MeRIP-Seq	25456834	GSE52662	GSM127370	GSM127370	mouse embr	PolyA+	Non-cancer	Mouse
ESC_BVSC	MeRIP-Seq	25569111	GSE61995	GSM151802	GSM151802	BVSC embry	PolyA+	Non-cancer	Mouse
iPSC	MeRIP-Seq	25683224	GSE52125	GSM126005	GSM126006	Induced plur	PolyA+	Non-cancer	Mouse
MEF	MeRIP-Seq	25569111	GSE61995	GSM151803	GSM151803	mouse embr	PolyA+	Non-cancer	Mouse
MSC	MeRIP-Seq	30429466	GSE114933	GSM315517	GSM315517	bone marrow	PolyA+	Non-cancer	Mouse
NSC_E12.5	MeRIP-Seq	25683224	GSE52125	GSM126005	GSM126006	E12.5-derive	PolyA+	Non-cancer	Mouse
NSC_E14.5	MeRIP-Seq	Citation missing	GSE104867	GSM280910	GSM280910	E14.5-derive	PolyA+	Non-cancer	Mouse
SC	MeRIP-Seq	25683224	GSE52125	GSM126005	GSM126006	Testicular se	PolyA+	Non-cancer	Mouse

Table S2. Validated m6A genes from literatures

Pubmed ID	Cell Line	GeneID	Gene Symbol	Species	Cell Name
24394384	mESC	66932	Rexo1	Mouse	embryonic stem cells
24394384	mESC	26424	Nr5a2	Mouse	embryonic stem cells
24394384	mESC	71950	Nanog	Mouse	embryonic stem cells
24394384	mESC	20674	Sox2	Mouse	embryonic stem cells
24394384	mESC	12591	Cdx2	Mouse	embryonic stem cells
24394384	mESC	14176	Fgf5	Mouse	embryonic stem cells
24394384	mESC	20664	Sox1	Mouse	embryonic stem cells
24394384	mESC	18508	Pax6	Mouse	embryonic stem cells
24394384	mESC	18012	Neurod1	Mouse	embryonic stem cells
24394384	mESC	20671	Sox17	Mouse	embryonic stem cells
24394384	mESC	14465	Gata6	Mouse	embryonic stem cells
24394384	mESC	14836	Gsc	Mouse	embryonic stem cells
24394384	mESC	16009	Igfbp3	Mouse	embryonic stem cells
27117702	A549	1956	EGFR	Human	lung cancer cells
27117702	A549	9261	MAPKAPK2	Human	lung cancer cells
27117702	A549	6901	TAZ	Human	lung cancer cells
27117702	A549	1788	DNMT3A	Human	lung cancer cells
27117702	A549	3265	HRAS	Human	lung cancer cells
27117702	A549	4609	MYC	Human	lung cancer cells
27590511	MCF-7	79923	NANOG	Human	breast cancer cells
27590511	MCF-7	9314	KLF4	Human	breast cancer cells
27919077	S2R+	42898	BRWD3	Drosophila	embryonic-derived cells
27919077	S2R+	31458	Usp16-45	Drosophila	embryonic-derived cells
27919077	S2R+	37233	Fak	Drosophila	embryonic-derived cells
27919077	S2R+	42445	H	Drosophila	embryonic-derived cells
27919077	S2R+	45398	Aldh-III	Drosophila	embryonic-derived cells
28017614	MONOMAC-6	51676	ASB2	Human	acute myeloid leukemia cells
28017614	MONOMAC-6	5467	PPARD	Human	acute myeloid leukemia cells
28017614	MONOMAC-6	5914	RARA	Human	acute myeloid leukemia cells
28017614	MONOMAC-6	6556	SLC11A1	Human	acute myeloid leukemia cells
28017614	MONOMAC-6	200081	TXLNA	Human	acute myeloid leukemia cells
28297667	GSC	8728	ADAM19	Human	glioblastoma stem cells
28297667	GSC	2042	EPHA3	Human	glioblastoma stem cells
28297667	GSC	9314	KLF4	Human	glioblastoma stem cells
28344040	GSC	2305	FOXM1	Human	glioblastoma stem cells
28792938	T cell	12703	Socs1	Mouse	T cell
28792938	T cell	12702	Socs3	Mouse	T cell
28792938	T cell	12700	Cish	Mouse	T cell
28869969	HSPC	30718	notch1a	Zebrafish	haematopoietic stem/progenitor cells
28869969	HSPC	394125	rhoca	Zebrafish	haematopoietic stem/progenitor cells
28920958	MOLM-13	4609	MYC	Human	acute myeloid leukemia cells
28920958	MOLM-13	596	BCL2	Human	acute myeloid leukemia cells
28920958	MOLM-13	5728	PTEN	Human	acute myeloid leukemia cells
28991227	GSC	6657	SOX2	Human	glioma stem-like cells
29171881	Huh-7	8835	SOCS2	Human	hepatocellular carcinoma cells
29171881	HepG2	8835	SOCS2	Human	hepatocellular carcinoma cells
29290617	MM6	4602	MYB	Human	haematopoietic stem/progenitor cells
29290617	MM6	4609	MYC	Human	haematopoietic stem/progenitor cells
29290617	NB4	4602	MYB	Human	haematopoietic stem/progenitor cells
29290617	NB4	4609	MYC	Human	haematopoietic stem/progenitor cells
29346752	DRG	11910	Atf3	Mouse	dorsal root ganglion cells
29346752	DRG	13197	Gadd45a	Mouse	dorsal root ganglion cells
29346752	DRG	14419	Gal	Mouse	dorsal root ganglion cells
29346752	DRG	16476	Jun	Mouse	dorsal root ganglion cells
29346752	DRG	17869	Myc	Mouse	dorsal root ganglion cells
29346752	DRG	20666	Sox11	Mouse	dorsal root ganglion cells
29346752	DRG	194388	Tet3	Mouse	dorsal root ganglion cells
29346752	DRG	22352	Vim	Mouse	dorsal root ganglion cells
29429926	MEF	11911	Atf4	Mouse	Mouse embryonic fibroblast cells
29547716	mESC	67302	Zc3h13	Mouse	Mouse embryonic stem cells
29573145	PBMC	27161	AGO2	Human	peripheral blood mononuclear cells
30046135	Hela	8408	ULK1	Human	cervical cancer cells
30065315	HSPC	21349	Tal1	Mouse	hematopoietic stem/progenitor cells
30065315	HSPC	14461	Gata2	Mouse	hematopoietic stem/progenitor cells
30065315	HSPC	12394	Runx1	Mouse	hematopoietic stem/progenitor cells
30065315	HSPC	20850	Stat5a	Mouse	hematopoietic stem/progenitor cells
30065315	hUCB HSC	3214	HOXB4	Human	umbilical cord blood hematopoietic stem cells
30065315	hUCB HSC	2309	FOXO3	Human	umbilical cord blood hematopoietic stem cells
30065315	hUCB HSC	861	RUNX1	Human	umbilical cord blood hematopoietic stem cells
30065315	hUCB HSC	4297	KMT2A	Human	umbilical cord blood hematopoietic stem cells
30065315	hUCB HSC	4089	SMAD4	Human	umbilical cord blood hematopoietic stem cells
30065315	hUCB HSC	2354	FOSB	Human	umbilical cord blood hematopoietic stem cells
30065315	hUCB HSC	6886	TAL1	Human	umbilical cord blood hematopoietic stem cells
30065315	hUCB HSC	7543	ZFX	Human	umbilical cord blood hematopoietic stem cells
30107516	N2A cells	14265	Fmr1	Mouse	Mouse neuroblastoma cells
30131850	HMEC	3725	JUN	Human	breast cancer cells
30131850	HMEC	6513	SLC2A1	Human	breast cancer cells
30131850	HMEC	1843	DUSP1	Human	breast cancer cells
30131850	HMEC	7428	VHL	Human	breast cancer cells
30150673	HSC	12443	Ccnd1	Mouse	hematopoietic stem cells
30150673	HSC	17869	Myc	Mouse	hematopoietic stem cells
30150673	HSC	12006	Axin2	Mouse	hematopoietic stem cells
30150673	HSC	19126	Prom1	Mouse	hematopoietic stem cells
30150673	HSC	12043	Bcl2	Mouse	hematopoietic stem cells
30150673	HSC	17210	Mcl1	Mouse	hematopoietic stem cells
30150673	HSC	12028	Bax	Mouse	hematopoietic stem cells
30150673	HSC	12015	Bad	Mouse	hematopoietic stem cells
30150673	HSC	12125	Bcl2l11	Mouse	hematopoietic stem cells
30150673	HSC	15205	Hes1	Mouse	hematopoietic stem cells
30150673	HSC	18128	Notch1	Mouse	hematopoietic stem cells
30154548	HEC-1-A	23035	PHLPP2	Human	endometrial cancer cells
30154548	HEC-1-A	55615	PRR5	Human	endometrial cancer cells
30154548	HEC-1-A	79899	PRR5L	Human	endometrial cancer cells
30154548	HEC-1-A	2475	MTOR	Human	endometrial cancer cells
30197299	mESC	232087	Mat2a	Mouse	embryonic stem cells
30305247	3T3-L1	890	CCNA2	Human	preadipocytes
30305247	3T3-L1	1017	CDK2	Human	preadipocytes
30428350	HepG2	5465	PPARA	Human	hepatocellular carcinoma cells
30429466	MSC	19228	Pth1r	Mouse	bone marrow mesenchymal stem cells
30463905	NHDF	3456	IFNB1	Human	normal Human dermal fibroblasts
30514900	OPC	50913	Olig2	Mouse	oligodendrocyte progenitor cells
30518868	NPEC	79755	ZNF750	Human	Nasopharyngeal carcinoma
30559377	MEF	15977	Ifnb1	Mouse	Mouse embryonic fibroblast cells
